# Development and Investigation of a Wearable Aid for a Load Carriage Task

**DOI:** 10.3390/ijerph17030749

**Published:** 2020-01-24

**Authors:** Saad A. Alabdulkarim, Abdulsalam M. Farhan, Mohamed Z. Ramadan

**Affiliations:** Industrial Engineering Department, College of Engineering, King Saud University, Riyadh 11421, Saudi Arabia; farhan002011@gmail.com (A.M.F.); mramadan1@ksu.edu.sa (M.Z.R.)

**Keywords:** assistive device, wearable aid, anterior load carriage, interventions

## Abstract

Anterior load carriage tasks are common and can lead to musculoskeletal disorders such as lower back pain. The objectives of this study were to develop a wearable carriage aid and examine its effectiveness on physical demands while considering the potential moderating influence of the carried load. The study consisted of two within-subject factors: device and load. For the former, two levels were tested: with and without the device worn. For the latter, two loads were examined: 15 and 30% of each individual’s body mass. Sixteen participants walked on a treadmill for five minutes at a constant speed for each condition. Physical demands were quantified using objective (EMG-based) and subjective (discomfort) measures. Wearing the device reduced static and median anterior deltoid, trapezius, and biceps brachii muscle activations. Increasing the carried load increased most physical demand measures. Two significant Device×Load interactions were observed; for the anterior deltoid and trapezius median activation measures, the influence of increasing load was lower when the device was worn. While slightly increasing perceived discomfort in the lower back, wearing the device reduced shoulder, neck, and hand/wrist discomfort. While the study demonstrated a potential for the device, future work is required under more realistic and diverse testing conditions.

## 1. Introduction

Despite the advancements in production technologies, manual material handling (MMH) tasks are still common in many occupational sectors [1,2]. This is potentially to benefit from worker experience, skill, and movement flexibility [3]. The solutions to address physically demanding tasks can be engineering or administrative ones. A common engineering solution is the use of automation. However, this approach can be unjustifiably expensive and/or practically infeasible. Automation costs can be even higher in developing countries where MMH tasks are more prevalent as human power remains a dominant procedure to perform occupational tasks [4,5,6]. Load carriage is a common occupational task and can be defined as any situation in which an additional load is added to the body [7], and can be seen as a mix of both lifting and pushing or pulling tasks [8]. Among all MMH tasks in the USA, Ciriello et al. [8] observed that carrying tasks were the third most frequent type of tasks and was preceded only by lifting and lowering tasks. Supporting this, carrying and lifting were identified as the most common physically demanding tasks performed by U.S. Army soldiers [9]. In a review study, Golriz and Walker [10] qualitatively synthesized evidence that carrying tasks can provoke lower back and shoulder pain. Van Vuuren et al. [11] completed an epidemiological study among a group of steel workers and observed a high association between load carriage tasks and lower back problems. These injuries can have economic effects. For example, the annual direct costs for shoulder and lower back injuries in the USA were estimated to be at least $7 billion [12] and $50 billion [13], respectively. The economic burden of musculoskeletal disorders (MSDs) is also substantial in other countries. For example, in Nordic countries, they cost ~3–5% of the gross national product [14,15], and in Canada, they cost ~3.4% of the gross domestic product [16]. In Great Britain, work-related MSDs led to an average of fifteen days away from work in 2013–2014 [17]. However, it should be noted that the magnitude of injury risks in the carrying tasks can be affected by several factors such as the carried load mass, size, and location [18,19,20,21] as well as personal factors such as gender [22,23] and age [18]. 

There are several approaches to perform a carrying task [24]. The most common method is the anterior load carriage (i.e., mounting objects in front of the body [24]). This particular procedure can have diverse effects. More specifically, an anterior load can require more muscular effort in contralateral muscles [25], can increase energy expenditure on shoulder muscles, and can increase in anterior/posterior shear loading of the spine when compared with other methods of carrying (posterior and lateral load carriage) [26]. This may suggest that this carrying approach poses greater risk for the back and shoulders injuries. Additionally, trunk extensors muscle activity has increased during an anterior load carriage task while walking compared with standing [27]. 

Carriage wearable aids are generally designed to transfer the carried load from the wearer’s hands to, for example, his/her shoulders and/or hip. This redistribution of the physical demands can also reduce external moment around the L5/S1. Few studies have specifically examined the effect of these devices on physical demands in carrying tasks. Muslim and Nussbaum [28] examined the effect of an upper-body wearable carrying aid on physical demands in a posterior load carriage (PLC) task while considering the moderating effect of the load mass and location; the aid was observed to reduce the lumbosacral moments in the heaviest load and highest location condition. This was seen as a potential for the device to reduce the risk of lower back pain in PLC tasks. Smallman, Graham, and Stevenson [29] studied the influence of an upper-body assistive device called a mover’s assistive device (MAD) on trunk-pelvis coordination. The MAD was worn like an upper-body armor. It increased the in-phase coordination between the trunk and pelvis, highlighting a potential mechanism by which the MAD may reduce injury risk. In a load carriage task while walking, Gregorczyk et al. [30] investigated the effect of a lower body wearable assistive device on metabolic demands and gait biomechanics. Compared to a control condition, the device increased metabolic demands and modified gait biomechanics regardless of the carried load. 

Overall, existing evidence suggests that there are inconsistent effects for carriage wearable aids on physical demands. Therefore, the objective of this study was to develop and investigate a low-cost wearable carriage aid in terms of physical demands while walking and while considering the potential moderating effect of load mass. Physical demands were defined using a comprehensive set of outcomes to ensure a more holistic evaluation. 

## 2. Materials and Methods 

### 2.1. The Aid Design Requirements and Development

**Figure 1:**
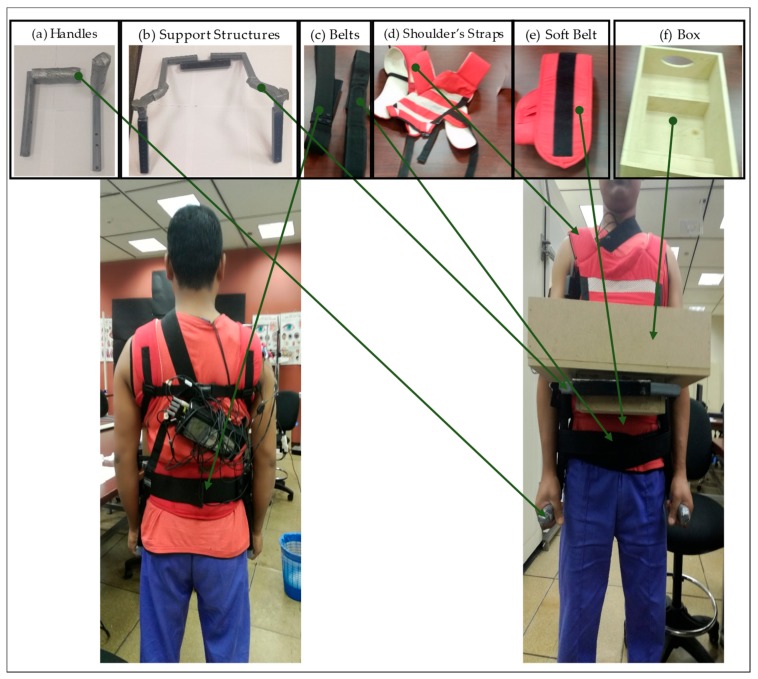
Components of the developed aid. Note that the strap close to the wearer’s neck was for the electromyography (EMG) data collection setup (i.e., the aid did not aim to load the neck). (**a**) shows the handles to be grasped while wearing the device. (**b**) shows the support structure that the box (**f**) goes into. (**c**) shows the belts that fix the device to the wearer’s body. (**d**) shows the shoulder straps to hold the device on the wearer’s body. (**e**) shows soft belts that set between the belts (**c**) and the wearer’s body. (**f**) shows the box where the carried loads can be put into.

The development of the aid underwent several iterations to reach a final prototype. Recall that in the traditional anterior load carriage, the person often holds the load with the hands below the carried load while flexing the shoulders. The designed aid was required to reduce demands on the lower back, shoulders, and hands. To achieve this goal, the final design (see Figure 1) aimed to redistribute the carried load demands over the wearer’s body. In this design, the shoulders, elbows, and wrists are in a neutral posture. These particular joints are often in a non-neutral posture in the traditional carriage. To potentially reduce hands demands, the load mass is supported by the upper back, shoulders, and waist straps. In other words, the load is resting on the noted body parts instead of mainly on the hands. 

**Figure 2:**
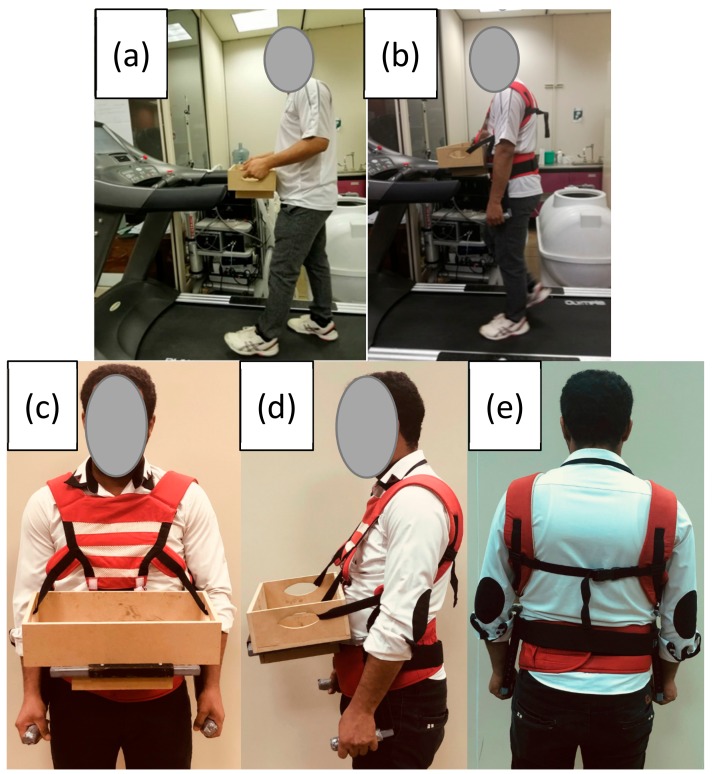
Pictures of a participant in the without (**a**) and with (**b**–**e**) assistive device.

The developed assistive device had four main connected parts: handles (Figure 1a), support structure (Figure 1b), hip belts (Figure 1c), shoulder straps (Figure 1d), soft belt (Figure 1e), and a box (Figure 1f). The purpose of the shoulder straps was to transfer the load to the upper back and shoulders. It connects the box to the individual shoulders and its length could be adjusted via Velcro straps. The contact area between the shoulder straps and the wearer’s body was a soft piece to reduce the potential pain that may result from the pressure of the load. Three hip belts were used in the developed assistive device; the soft belt (Figure 1e) maintained direct contact with the waistline of the wearer. This belt was made of a comfortable soft-padded lightweight cloth (sponge). The other two belts (Figure 1c) were made of a relatively strong material and were for maintaining the load balance around the hip. The three belts were adjustable via Velcro straps. Two identical pieces of metal (Figure 1b) formed the supporting structure. Each piece was an L-shaped tube to be connected to a perforated square tube. Two pins were used to hold each of the L-shaped pieces to the perforated square tube. Adjustable L-shaped handles (Figure 1a) fit in the arms and were locked by pin in a suitable position depending on the individual’s anthropometry. The goal here was to maintain the noted posture neutrality. The box (Figure 1f) was specially designed box to be inserted in the support structure. The carried masses were placed inside this box. Overall, the aid weighed 2.5 kg. It was desired to minimize the device weight to the extent possible to reduce the resulted physical demands. Figure 2 shows more pictures of a person wearing the developed device.

### 2.2. Participants

Sixteen male participants were recruited for this experiment. The sample size was determined using the effect size (partial eta-squared: $\eta_{p}^{2})$ for an important dependent measure (peak moment around the L5/S1) in a similar study [31]. Using the found $\eta_{p}^{2}$ of 0.088, a power of 0.8, and Type I error of 0.05, the study required sixteen participants as determined by G*Power software [32].

Table 1 shows anthropometric measures for the sample. All participants reported being physically active and having no history of lower back pain, upper/lower limbs problems, nor any medical conditions that might affect the results or put the participants under high injury risk. Participants signed an informed consent form that was approved by the Human Participants Review Sub-committee of the Institutional Review Board (IRB) of King Saud University, College of Medicine, and King Khalid University Hospital (research project # E-18-3533). All participants were compensated by a symbolic amount of money for their time ($13/h). 

### 2.3. Task Description

The task was an anterior load carriage. The task was performed while walking on a treadmill at a constant speed (2 km/h) for five minutes. These conditions were assumed to represent somewhat practically relevant conditions and were used in similar studies [33,34]. Additionally, pilot testing was conducted to ensure that participants could complete the task under all testing conditions. 

### 2.4. Independent Variables

The experiment included two within-subject factors: Assistive Device and Carried Mass. For the former, there were two conditions (With and without the device). For the latter, two masses were examined to see if the potential effectiveness of the assistive device depended on the carried masses. The amount of carried mass was set relative to each participant’s body mass; the examined levels were Light (15%) and Heavy (30%). In the pilot test, participants were confirmed as capable of completing the task particularly in the Heavy condition. Increments of 3-Kg were used to modify the carried load mass. The same box was used in both Assistive Device conditions (Figure 2). 

### 2.5. Procedures and Data Collection

A repeated measures design was used. In this design, participants performed the task in all four combinations of Assistive Device and Carried Mass. The study was completed in a laboratory and in a single session that lasted for ~2.5 h. The order of exposure to the four conditions was counterbalanced using 4 × 4 Balanced Latin Squares. At least 10 min of rest was provided between conditions to minimize any influences from residual muscle fatigue. Participants were encouraged to ask for more rest as needed. 

At the start of the session, testing conditions were explained to the participants. They subsequently signed the informed consent and their anthropometric measurements were collected. To be more familiar with the treadmill walking on the noted speed (2 km/h), participants practiced walking on the treadmill for ~5 min [35,36]. After that, Borg’s 10-point scale [37] was explained to the participants to be able to report ratings of perceived discomfort (RPDs) for several body parts (see the Dependent Measures subsection). To help understand the noted scale, as well as to normalize ratings over the whole range, participants were asked to perform a static endurance task (leaning against a wall with the knees bent at ~90°) and provide RPDs for the thigh area every 5 s until reaching maximum discomfort [38,39,40,41]. 

Subsequently, surface electromyography (EMG) electrodes were placed over the following four muscles: the dominant side (all participants were right-handed) of the flexor digitorum profundus, biceps brachii, anterior deltoid, trapezius and both sides of the erector spinae muscle. These particular muscles were selected because they were accessible in all conditions and were recruited in the examined task. Prior to the electrode placement, the skin was shaved, lightly abraded, and cleaned using 70% alcohol. Pairs of bipolar Ag/AgCl electrodes with a 2.5 cm inter-electrode distance were subsequently placed on the skin as recommended by Hermens et al. (1999) [42]. Raw EMG signals were collected at 1000 Hz using the ME6000 System (Mega Electronic, Kuopio, Finland). 

Subsequently, participants performed three trials of maximum voluntary contractions (MVCs). Nonthreatening verbal encouragement was provided during the MVC trials. At least one min of rest was provided between these trials. At least 10 min of rest was provided at the end of all MVCs. 

Participants then performed the testing trials following the noted counterbalancing procedure. Before starting a condition with the device, the experimenter assisted the participant in wearing the device. A brief practice (~2 min) was provided on the treadmill while wearing the device to ensure a comfortable fit of the device on the wearer. At the end of each testing condition, participants provided RPDs of several body parts. 

### 2.6. Dependent Variables

Objective and subjective measures of physical demands were collected. For the former, muscular activities were measured through EMG. The activity of some important muscles recruited in the task was monitored. These muscles were the trapezius and the anterior deltoid from the shoulder, the right and left sides of the erector spinae from the lower back, the biceps brachii from the upper arm, and flexor digitorum profundus from the lower arm. The raw EMG data for all investigated muscles were band-pass filtered (20–500 Hz) [43]. Subsequently, EMG root mean square (RMS, time constant 1000 ms) were calculated over the testing duration (5 min) and these were subsequently normalized (nEMG) to maximal RMS values found in the MVC trials following the procedure explained in [43]. The initial five seconds were removed from the investigation to avoid the interruption of starting, as conducted in [44]. From the nEMG data, the 10th and 50th percentile values were determined for each condition, and were considered representative of the static and median loadings, respectively [45]. Ratings of perceived discomfort (RPDs) experienced in the neck, shoulders, lower back, and wrist/hands were collected using Borg’s 10-point scale [37]. These data were collected immediately after each condition was completed.

### 2.7. Statistical Analysis of Data

Separate 2 × 2 repeated measures analyses of variance (ANOVA) were performed for each dependent variable to assess the effects of Assistive Device and Carried Mass. Parametric model assumptions were tested, and data transformation were done as needed to meet these assumptions. Conditions presentation order factor was not found significant on any dependent measure. Post-hoc comparisons were performed using simple-effects tests. Partial eta-squared ($\eta_{p}^{2}$) was calculated to quantify the effect sizes. All statistical tests were considered significant when *p* < 0.05. Minitab statistical software (Minitab v.18, Minitab Inc., State College, PA, USA) was used for the statistical analyses and Matlab 2015a (The MathWorks Inc., Natick, MA, USA) was used for data processing. 

## 3. Results

### 3.1. Median Loading Metrics (50th Percentile nEMG)

**Figure 3:**
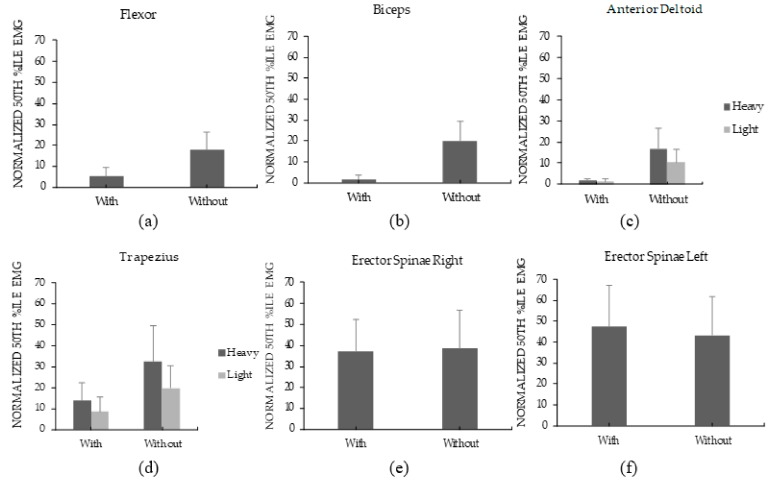
Percentages of the mean values of 50th %ile normalized EMG (nEMG) for the With and Without Device conditions for Flexor (**a**), Biceps (**b**), Anterior Deltoid (**c**), Trapezius (**d**), Erector Spinae R (**e**), and Erector Spinae L (**f**). Error bars represent standard deviations. Means were calculated across the two Load levels except for figures c and d given the significant Device×Load interactions.

The mean median measures across the two Load levels and across all tested muscles were 17.4 and 26.5% of MVC in the with and Without device conditions, respectively. More specifically, using the device significantly reduced the median loading measures of all tested muscles except for the bilateral measures of Erector Sp (Table 2). Among these particular muscles, the magnitude of reduction ranged from 56.7% in the trapezius to 91.8% in the biceps (Figure 3). Generally, increasing the Load significantly increased most median activity measures. There were two significant Device×Load interactions (Table 2); the influence of increasing the Load on both the trapezius and anterior deltoid was lower in the with device condition (Figure 3).

### 3.2. Static Loading Metrics (10th Percentile nEMG)

**Figure 4:**
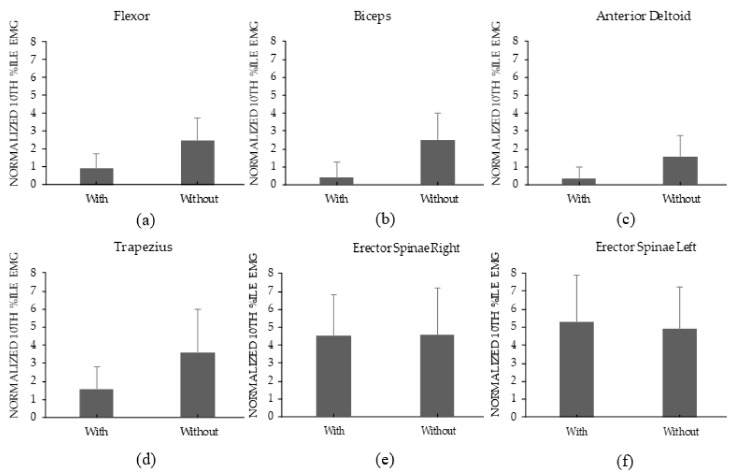
Percentages of the mean values of 10th %ile nEMG for the With and Without Device conditions for Flexor (**a**), Biceps (**b**), Anterior Deltoid (**c**), Trapezius (**d**), Erector Spinae R (**e**), and Erector Spinae L (**f**). Error bars represent standard deviations. Means were calculated across the two Load levels.

Across the two Load levels and all examined muscles, the mean static loading measures were 2.1 and 3.2% of MVC in the with and without device conditions, respectively. Similar to the median loading results, using the device significantly reduced the static loading of most tested muscles except the bilateral measures of the Erector Sp (Figure 4). Among these specific muscles, the amount of reduction ranged from 56.2% in the trapezius to 84.1% in the deltoid. Increasing the Load significantly increased static muscle activity of the trapezius and the bilateral measures of the Erector Sp (Table 3). Influences of Load were consistent across levels of Device (i.e., no significant Device×Load interactions were found here).

### 3.3. Rating of Perceived Discomfort

**Figure 5:**
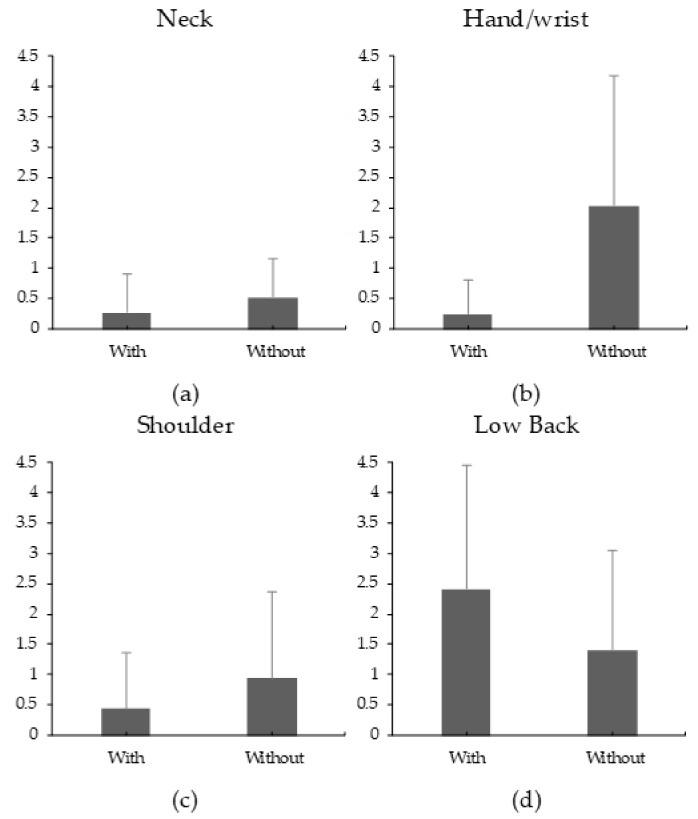
Mean ratings of perceived discomfort (RPD) for the neck (**a**), Hand/wrist (**b**), Low Back (**c**), and Shoulder (**d**). The Borg CR-10 scale was used to obtain these ratings. Error bars represent the standard deviation for each mean.

Across the two Load levels and all monitored body parts, the mean RPD ranged from 0.25 (in the Hand/wrist) to 2.4 (in the Low Back) in the With and Without device conditions, respectively. While increasing the Low Back RPD, using the device significantly reduced the RPD of the Neck, Shoulders, and Hand/wrist (Figure 5). Increasing the Load significantly increased RPD of all monitored body parts, regardless of Device condition (Table 4).

## 4. Discussion

The main objective of this study was to examine the effectiveness of a low-cost wearable device for carriage task while considering the potential moderating influence of the carried mass. The investigation was conducted for a load carried anteriorly in five-minute walk on a treadmill. Overall, using the device led to lower physical demands (RPDs and both median and static muscle loading measures) and this was somewhat consistent across the two Load levels. 

Using the device led to a lower median and static muscle loading of the flexor, biceps, anterior deltoid, and trapezius (Figure 3 and Figure 4). In line with these results, wearing the device also reduced the RPD of the neck, hands/wrist, and shoulder (Figure 5). Although no motion capture system was used to monitor posture, wearing the device apparently changed the posture needed to complete the carriage task to a more neutral one. This change potentially led to the noted reduction in muscle activation. Supporting this, Anderson et al. (2007) showed that anteriorly carrying a load at knuckle height can reduce both biceps and anterior deltoid muscle activities [27]. Additionally, maintaining a neutral posture for the arms and hands can reduce the moment arms and shear forces that affect the lower back during the carriage task [26].

While the device reduced upper body demands, it slightly increased lower back demands as seen in the Low Back RPD (Figure 5d). To demonstrate that this increase was marginal, wearing the device did not affect the median and static bilateral measures of Erector Sp (Figure 3 and Figure 4). A similar tradeoff between the upper body and lower back demands was observed when using an upper body passive assistive device for a simulated overhead task [40]. The increase in lower back demands was potentially to counteract the external moment generated by the carried load around the lower back. The tradeoff between body parts is a common challenge in assistive devices design (i.e., when reducing demands on a certain body part, demands on different regions increase). Another tradeoff was observed in a simulated assembly task: a passive assistive device increased chest discomfort while increasing leg and back demands [46].

The developed device has the potential to reduce the influence of increasing the carried load mass. This was supported by the two significant Load×Device interactions on the anterior deltoid and trapezius median activation measures (Table 2). The effect of increasing the load was less influential on the noted measures when the device was worn. This benefit can be explained by the described change of posture when the device was worn (discussed earlier) and the redistribution of the carried load over the wearer’s body. The rest of the physical demands measures did not indicate significant Load×Device interactions. Supporting this, Muslim and Nussbaum (2016) observed the effect of a posterior carrying aid on several regional RPD measures to be independent of the carried load [28]. 

Several limitations in the study have to be recognized. While performing the study in a laboratory environment ensured relatively high internal validity, the extent of the external validity is unknown. This is particularly important given the short testing duration (five minutes for each condition). Whether the results obtained here reflect long-term outcomes or not is unclear. Also, all recruited participants were males. It is unknown whether females will have the same pattern of results or not. Additionally, only two levels from the Load factor were tested. This design prevents detecting any non-linearity in the Device×Load relationship. However, this limitation should not be critically concerning because the study found only two significant Device×Load interactions. There are other aspects were not examined here. Among these, the effect of the device on postural balance was not quantified here. Postural control needs to be evaluated during walking and upright stance to fully understand the effects of wearing the device on slip, trip, and falls risks [47]. Additionally, it is practically important to examine/improve the donning/doffing requirements of the device. 

## 5. Conclusions

Despite the noted limitations, this study highlighted the potential for a low-cost wearable assistive device to reduce physical demands (perceived discomfort for several body regions and both static and median muscle activations) in anterior carriage tasks. However, wearing the device slightly increased lower back RPD. The effectiveness of the device was independent of the carried load except for the anterior deltoid and trapezius median activation measures. For these particular measures, the influence of increasing load was lower when the device was worn. Insights from this study can be used to develop a better design for the wearable aid. Since the device has slightly increased lower back demands, an updated design should be developed to better balance the weight of the carried load around the wearer body and/or transfer the load to the ground through leg structures as done in the FORTIS^TM^ (Lockheed-Martin, Bethesda, MD, USA) exoskeleton [48]. Future research is needed to examine the developed device under more diverse and realistic conditions and under longer testing durations. 

## Figures and Tables

**Table 1:** Participants’ anthropometrics.

Measurement	Mean (SD)
Age (year)	34.63 (5.54)
Weight (kg)	69.97 (7.85)
Height (cm)	164.66 (4.89)
Waist Circumference (cm)	91.81 (6.13)
Body Breadth (cm)	49.34 (3.26)
Waist Height (cm)	91.97 (3.66)
Knuckle Height (cm)	70.74 (3.47)

**Table 2:** Statistical results for the main and interactive influences of the device and load on median loading measures. Any transformation performed to the dependent measures are indicated. *P* values are indicated with effect sizes ($\eta_{p}^{2}$) in parentheses. Bold font highlights significant effects (*p* < 0.05).

Response	Trans.	Device	Load	Device × Load
Flexor		**<0.0001** (0.73)	**0.003** (0.18)	0.21 (0.04)
Biceps		**<0.0001** (0.77)	0.05 (0.08)	0.18 (0.04)
Anterior Deltoid		**<0.0001** (0.70)	**0.004** (0.17)	**0.01** (0.13)
Trapezius		**<0.0001** (0.61)	**<0.0001** (0.37)	**0.04** (0.09)
Erector Sp. R	(Ln)	0.79 (0.00)	**<0.0001** (0.48)	0.32 (0.02)
Erector Sp. L		0.10 (0.06)	**<0.0001** (0.44)	0.91 (0.00)

**Table 3:** Statistical results for the main and interactive influences of the Device and Load on static loading measures. Any transformation performed to the dependent measures are indicated. *p*-values are indicated with effect sizes ($\eta_{p}^{2}$) in parentheses. Bold font highlights significant effects (*p* < 0.05).

Response	Trans.	Device	Load	Device × Load
Flexor		**<0.0001** (0.52)	0.05 (0.09)	0.63 (0.01)
Biceps	(Ln)	**<0.0001** (0.64)	0.59 (0.01)	0.96 (0.00)
Anterior Deltoid		**<0.0001** (0.52)	0.13 (0.05)	0.08 (0.07)
Trapezius		**<0.0001** (0.53)	**<0.0001** (0.30)	0.12 (0.05)
Erector Sp. R		0.40 (0.02)	**<0.0001** (0.45)	0.15 (0.04)
Erector Sp. L		0.20 (0.04)	**<0.0001** (0.39)	0.09 (0.06)

**Table 4:** Statistical results for the main and interactive influences of the Device and Load on ratings of perceived discomfort (RPD) measures. Any transformation performed to the dependent measures are indicated. *P* values are indicated with effect sizes ($\eta_{p}^{2}$) in parentheses. Bold font highlights significant effects (*p* < 0.05).

RPD	Trans.	Device	Load	Device × Load
Neck	Sqrt	**0.014** (0.126)	**0.039** (0.091)	0.865 (0.001)
Shoulders	Sqrt	**0.025** (0.107)	0.094 (0.061)	0.702 (0.003)
Low Back		**0.012** (0.131)	**0.027** (0.104)	0.419 (0.014)
Hand/wrist		**<0.0001** (0.383)	0.170 (0.041)	0.170 (0.040)
